# Cold Induced Sweating Syndrome with Urinary System Anomaly Association

**DOI:** 10.1155/2013/173890

**Published:** 2013-08-29

**Authors:** Salim Aljabari, Emily Howard, Todd Bell, Tetyana L. Vasylyeva

**Affiliations:** Department of Pediatrics, Texas Tech University Health Science Center, Amarillo, TX 79106, USA

## Abstract

Mutation in the cytokine receptor-like factor 1 and the cardiotrophin-like cytokine (*CRLF1* or *CLCF1* genes) phenotypically presents as cold induced sweating syndrome (CISS), which is a rare autosomal recessive disorder. The syndrome is characterized by paradoxical sweating in cold weather, dysmorphic facial features, musculoskeletal deformities, difficulty in feeding, and unexplained recurrent episodes of high-grade fever. We are presenting the first case of CISS with urinary system anomaly, which might relate to CRLF1/CLCF1 complex role in the embryonal nephrogenesis.

## 1. Introduction

Cold induced sweating syndrome (CISS; MIM number 272430) was first described by Soher et al. (1978) when they reported two siblings from Israel with paradoxical cold induced sweating and dysmorphic features [1]. Crisponi reported 17 newborns he encountered over 25 years ago from Sardinia, Italy, with characteristic dysmorphic facial features, musculoskeletal deformities, difficulty feeding, and unexplained recurrent episodes of high-grade fever; he suggested an autosomal recessive inheritance pattern, this was later called the Crisponi syndrome (CS; MIM number 601378) [2]. It was found that both CISS and CS were caused by mutation in the same gene; the cytokine receptor-like factor 1 (*CRLF1)* [3, 4]. Over the last 10 years it has been proven that CS and CISS represent a spectrum of one disorder with some differences in the clinical features between infancy and childhood rather than two entities [5–7]. 

The majority of the reported cases were from Turkey, and Italy; few from Israel, Norway, Australia, and Canada, none of the previously reported cases had association with urinary system anomalies although CRLF1/CLCF1 complex was found to be involved in the embryonal nephrogenesis [8–12]. 

Because CISS is a rare syndrome with very few reported cases in the literature, the full spectrum of clinical features of the syndrome is yet to be elucidated. We are presenting the first patient with confirmed CISS and urinary tract anomaly association.

## 2. Case Report

A 16-year-old Hispanic male patient was seen at the pediatric nephrology clinic for evaluation of a solitary left kidney. His past medical history was significant for difficulty in feeding during infancy when he required gastrostomy tube. He also had bilateral contracture of the elbow joints and right foot valgus deformity which was diagnosed with arthrogryposis, language developmental delay, bilateral campylodactyly, scoliosis, and left side cataract which later was corrected surgically.

During the office visits the patient was noticed to have excessive sweating. It was found that the patient had been having excessive sweating for a long time, primarily from the trunk, and surprisingly sweated only when he was cold. The family medical history and the birth history were noncontributory. The patient has a healthy sister and no affected family members.

**Figure 1:**
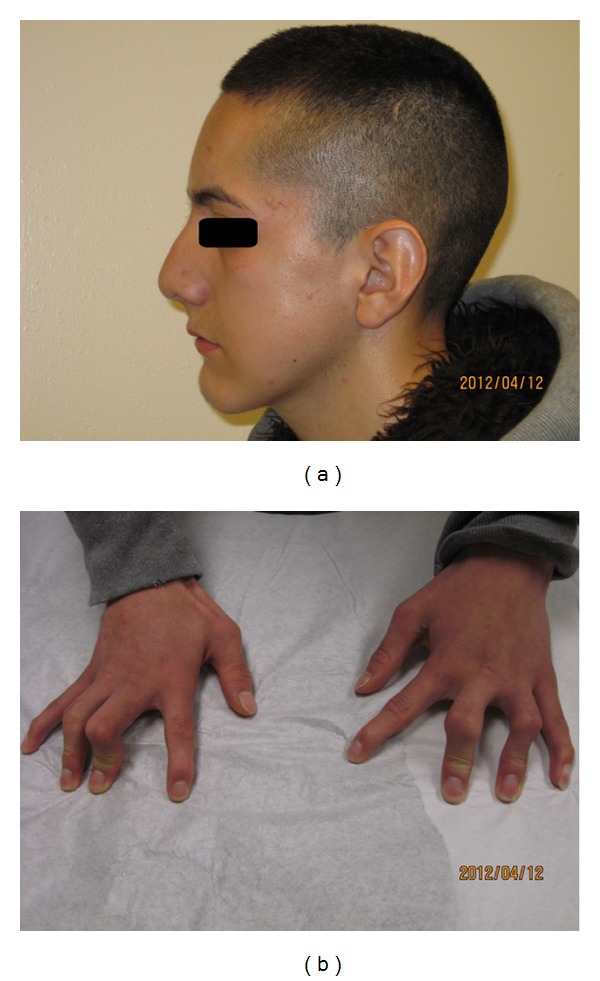
Pictures of the patient showing: (a) facial dysmorphic features and (b) campylodactyly.

On physical examination, the patient's weight was in the 5th percentile and the height in the 10th percentile for his age. Vital signs including blood pressure were within normal limits. Parental consent was obtained before photographs were taken. Patient head was normocephalic but with dysmorphic features (hypertelorism, short wide neck, large pinna, broad nasal bridge, and tented palate) (Figure 1(a)), and he had opacification of both corneas. Musculoskeletal exam revealed scoliosis, contracture around both elbows with limitation of movement, and bilateral campylodactyly (Figure 1(b)) and normal neurological exam was revealed with no focal neurological deficit. Evaluation of serum electrolytes revealed mild persistent hypernatremia.

**Figure 2:**
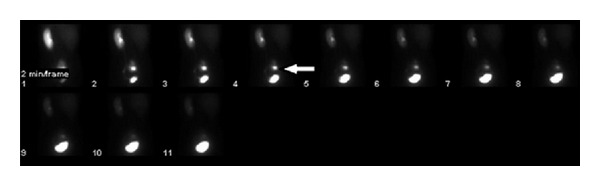
MAG 3 renal scan showing the small right kidney (the arrow) in the pelvis.

MAG-3 scan showed small right ptotic kidney in pelvis with normal blood flow, configuration, and excretion, with normal left kidney which had the majority of functions (Figure 2). Sweat-map testing showed excessive sweating from the waist upwards in a diffused pattern. 

Gene sequencing was performed in Norway (Dr. Boman laboratory) and showed that the patient is homozygous for *CRLF1* mutation, c.713dupC. 

The patient was treated with Clonidine which resulted in a dramatic decrease of cold induced sweating within 3-4 days.

## 3. Discussion

Two different mutations can cause CISS; *CRLF1* on chromosome 19 is the most common (90%), it gives CISS type 1 and *CLCF1* on chromosome 11 (10%) gives CISS type 2; both types are phenotypically indistinguishable, and they have the same sequence of events [4, 13, 14]. 

Patients with CISS have characteristic dysmorphic features (high arched palate, depressed nasal bridge, chubby cheeks, micrognathia, campylodactyly, and muscle contractures especially around the elbows). During infancy the most disabling feature is their tendency to startle; they have frequent episodes of severe facial muscle contractions usually in response to tactile stimuli, feeding, or crying; those episodes are severe enough to cause apnea and cyanosis, lead to difficulty in feeding and failure to thrive where most of the patients require gastrostomy tube for feeding. Patients also experience recurrent episodes of high-grade fever with no evidence of infection, explained by poor thermal regulation [2, 15].

From the 17 patients Crisponi presented in his paper, only 2 survived beyond the first two years of life this can be explained by the nutritional difficulties or the episodes of apnea; classically the episodes of facial contractions and the high-grade fever disappear completely by the second year of life, and then patients start developing progressive kyphoscoliosis and other musculoskeletal deformities, usually severe enough to require orthopedic surgeries [8].

Excessive sweating is the most disabling and persistent symptom beyond infancy; characteristically they sweat excessively when they are cold and mainly from the trunk; and usually have very little sweating when the temperature is high, making them uncomfortable in the warm weather, although the precise mechanism is unknown; the cold induced sweating can be successfully treated with Clonidine and Moxonidine, most likely by reducing the Noradrenaline production in the central nervous system [9]. Growth delay, developmental delay, decrease pain sensation, and hyperintense lesions in the subcortical white matter on the brain MRI were reported with some of the patients, basic hematologic and biochemical workup usually does not show any abnormalities [5, 8, 14, 16].

CLCF1 protein must combine with CRLF1 protein to form a complex that can be excreted from the cells; then the active complex of the two proteins activates a membrane bound receptor called the ciliary neurotrophic factor receptor (CNTFR), which is expressed abundantly on the central and the peripheral nervous systems cells during the embryonic life [14, 17, 18]; this possibly explains the paradoxical sweating phenomenon. Expression of CNTFR in the embryonic muscle tissues [6, 19, 20] and CLCF1 and CRLF1 deficiency during development most likely link to the abnormal muscle contractions observed in the patient, the orofacial muscle hypotonia, and the other neuromuscular features that CISS patients have [21, 22]; CNTFR also has been found to play a role in bone formation and metabolism [21, 22] leading to the musculoskeletal deformities patients with CISS have at birth.

The patient we are presenting had small ectopic nonfunctioning right kidney in the association with CISS. The recent studies have shown the important role which CRLF1/CLCF1 complex plays in the genitourinary system development in the embryonic life [10–12]. The ureteric bud tip is rich with CRLF1/CLCF1 complex which acts as a potent inducer of nephrogenesis [11]. Although it is still not understandable why only the one kidney was affected, we might speculate that codevelopment with other organs (spinal cord, skeletal anomalies) played a role. 

## 4. Conclusion 

Urinary anomaly is an additional feature that CISS patients may have, which can be at least in part explained by the role of CLCF1/CRLF1 in the embryonal nephrogenesis. The syndrome might be misdiagnosed as arthrogryposis and should be consider in patients with massive skeletal malformations. Early diagnosis and treatment of this syndrome can improve the quality of life of those patients.
